# The *C. elegans* gene *pan-1* encodes novel transmembrane and cytoplasmic leucine-rich repeat proteins and promotes molting and the larva to adult transition

**DOI:** 10.1186/1471-213X-13-21

**Published:** 2013-05-17

**Authors:** Chris R Gissendanner, Tram Do Kelley

**Affiliations:** 1 Department of Basic Pharmaceutical Sciences, College of Pharmacy, University of Louisiana at Monroe, Monroe, LA 71209, USA; 2 Department of Biology, University of Louisiana at Monroe, Monroe, LA 71209, USA

**Keywords:** *pan-1*, Leucine-rich repeat, Gonad, Vulva, Molting, Heterochronic, *lin-29*

## Abstract

**Background:**

Extracellular leucine-rich repeat (eLRR) proteins are a highly diverse superfamily of membrane-associated or secreted proteins. In the membrane-associated eLRR proteins, the leucine-rich repeat motifs interact with the extracellular matrix and other ligands. Characterizing their functions in animal model systems is key to deciphering their activities in various developmental processes.

**Results:**

In this study, we identify *pan-1* as a critical regulator of *C. elegans* larval development. *pan-1* encodes both transmembrane and cytoplasmic isoforms that vary in the presence and number of leucine-rich repeats. RNAi experiments reveal that *pan-1* is required for developmental processes that occur during the mid to late larval stages. Specifically, *pan-1* loss of function causes a late larval arrest with a failure to complete development of the gonad, vulva, and hypodermis. *pan-1* is also required for early larval ecdysis and execution of the molting cycle at the adult molt. We also provide evidence that *pan-1* functionally interacts with the heterochronic gene *lin-29* during the molting process.

**Conclusions:**

We show that PAN-1 is a critical regulator of larval development. Our data suggests that PAN-1 promotes developmental progression of multiple tissues during the transition from a larva to a reproductive adult. We further demonstrate that the activity of PAN-1 is complex with diverse roles in the regulation of animal development.

## Background

The leucine-rich repeat (LRR) is a common protein-ligand interaction motif found in a variety of functionally distinct proteins in both prokaryotes and eukaryotes [1]. Amongst the LRR-containing proteins is the structurally diverse superfamily of extracellular leucine-rich repeat (eLRR) proteins. These proteins are transmembrane, GPI-linked, or secreted and are characterized by having multiple LRR motifs in the extracytoplasmic domain [2]. Even though eLRR proteins are highly diverse, they can be grouped into subfamilies of related proteins based on the number and sequence of LRR motifs and the presence of other protein domains [3,4]. The LRR motif contains a conserved N-terminal consensus sequence LxxLxLxxN/CxL where ‘x’ is any amino acid and ‘L’ is typically Leu but can also be Ile, Val, or Phe [1]. The C-terminal region of the repeat is variable in the number and sequence of amino acids. Structural studies have shown that LRRs form a horseshoe-shaped structure with a hydrophobic concave surface formed by the leucine-containing β-strands and a convex surface containing the variable region with the hydrophobic core flanked by distinct N-terminal and C-terminal capping domains (LRR-NT and LRR-CT, respectively) [5,6].

Four groups of eLRRs have been identified based on hierarchical clustering [3]. These include eLRRs associated with immunoglobin and/or fibronectin type III domains (the ‘LRR_Ig/FN3’ group); a group containing the toll-like receptors (TLRs) or other eLRRs that group with the TLRs (‘LRR_Tollkin’ group); a group of eLRR proteins that do not contain other recognizable domains (‘LRR_Only’ group); and a group of eLRR proteins associated with a variety of other domains, including a large number with a G protein-coupled multi-transmembrane domain (‘LRR_Other’). In addition, there many eLRR proteins that cannot be clustered based on their structural characteristics (‘LRR_Only singletons’). Within each group there exists both structural and functional variety. Many eLRR proteins, however, have well-characterized neuronal functions and are associated with human neurological conditions [7]. Extracellular matrix organization is another cellular function associated with several eLRR proteins, especially the small leucine-rich repeat proteoglycans (SLRPs) [8]. Cell signaling functions of eLRR proteins are less well-characterized, although a number are known to modulate other signaling pathways [9-11].

Outside of the TLRs, and other eLRR proteins that exhibit clear homology to mammalian proteins, few eLRR proteins have been functionally characterized in the fruit fly and worm model systems. Nonetheless, studies to date in these systems indicate that divergent eLRR proteins are critical components of developmental processes. Capricious (Caps) and Tartan (Trn), for example, regulate cellular interactions that underlie boundary formation and tissue morphogenesis in *Drosophila*[12,13]*.* Caps also has a role in synaptic specificity [14,15]. In *C. elegans,* the ‘LRR-only’ proteins LET-4, EGG-6, and SYM-1 maintain epithelial integrity and are necessary for development of the excretory system [16]. Another ‘‘LRR-only” protein, DMA-1, promotes formation of dendritic arbors [17].

The *C. elegans* eLRR protein PAN-1 (P-granule Associated Novel protein 1) is another divergent eLRR protein with novel functions. PAN-1 was identified in screen for genes that encode proteins that physically interact with the germline RNA helicase protein GLH-1 [18]. PAN-1 co-localizes with P-granules and is essential for reproduction and larval growth. *pan-1* has also been identified in other previous genome-wide studies or screens, including an RNAi screen for genes that are required for molting [19]; and as a gene up-regulated in *unc-95* mutants [20]. *unc-95* encodes a LIM domain-containing protein that is expressed in body wall muscle [21].

Here, we report the identification of the *pan-1* gene in an RNAi screen for genes required for development of the spermatheca, a somatic gonad organ that functions in ovulation and sperm storage. One isoform of PAN-1, PAN-1B, is a predicted type I transmembrane protein that contains 15 eLRRs but lacks other conserved motifs or domains. In addition, we show that the *pan-1* gene also encodes likely cytoplasmic isoforms of the protein. We show for the first time that in the absence of *pan-1* function the developmental progression of multiple tissues are disrupted in the larva to adult transition, including the somatic gonad, vulva, and hypodermis. We find that *pan-1* is necessary for executing the L4 to adult molting process and functionally interacts with the heterochronic gene *lin-29* at the L4 to adult molt. This study increases the repertoire of developmental functions associated with eLRR proteins and provides a unique biological framework to dissect eLRR protein activity and reveal potentially novel eLRR protein-mediated signaling processes.

## Results

### Identification of spermatheca development genes

We performed an RNAi screen designed to identify genes required for development of the spermatheca. The screen was focused to chromosome III and composed of two-parts. The first part identified genes with loss of function phenotypes that included reduced brood sizes/sterility, and/or abnormal egg morphology. These phenotypes are associated with defects in spermatheca development [22]. This initial screen identified 190 genes (out of 2,402 genes in the Chromosome III library) that displayed reproductive loss of function phenotypes. A comparison of our results to a list of Chromosome III genes at wormbase.org that were annotated to have a sterile progeny (STP) phenotype revealed an overlap of only 20 genes. This indicated that our screen was identifying new reproductive genes. The discrepancy between our data set and wormbase.org is likely due to our use of an RNAi-sensitized stain (GR1373 (*eri-1 (mg366)*) as well as our procedure of observing adults derived from RNAi treated L1 larvae (a single generation screen) in contrast to observing adult progeny of L4 treated animals (a two generation screen). To ensure the most robust and comprehensive gene set, we repeated the RNAi experiments in triplicate on our gene set and the non-overlapping wormbase.org set. This gave a final set of 110 reproductive genes (92 repeats from our gene set and an additional 18 genes from the non-overlapping Wormbase set).

These genes were then re-screened by RNAi using a spermathecal marker, AJM-1::GFP, which is localized to the adherens junctions of spermathecal cells [23,24]. The secondary screen identified 31 genes with abnormal spermatheca development RNAi phenotypes (Additional file 1: Table S1). RNAi phenotypes of spermatheca development genes fell into two classes. One class exhibited AJM-1:GFP expression in the spermatheca but the pattern was abnormal indicating defective morphogenesis of the organ. A normal spermatheca at the young adult stage has an elongated conical shape with a proximal-distal polarity with distal cells being smaller. AJM-1::GFP localization outlines ordered rows of cells surrounding a central lumen (Additional file 2: Figure S1A, B). In RNAi animals with defective spermatheca morphogenesis, there is lack of proximal-distal polarity and the spermathecal cells exhibit a disorganized arrangement (Additional file 2: Figure S1C, D). Animals in the other class lack AJM::GFP expression in the spermatheca region of the gonad (Additional file 2: Figure S1E, F). This pattern indicates that a spermatheca was not formed or exhibits severe differentiation defects. Most of the spermatheca development genes identified in our screen appear to have either cellular “housekeeping”-type functions or are involved in mitosis or cytokinesis (Additional file 1: Table S1). Two genes, *C23G10.8* and *ZC395.4*, did not have any recognizable protein domains or homology outside of nematodes. One gene, *C16A3.4*, encodes a broadly conserved but uncharacterized C_2_H_2_-type zinc finger protein. Two genes, *ttr-32* and *pan-1,* encode apparent nematode-specific proteins but contain a conserved domain (transthyretin-like domain for TTR-32) or motif (leucine-rich repeat for PAN-1). *pan-1* was selected from this screen for further characterization.

### Sequence characterization of *pan-1*

**Figure 1:**
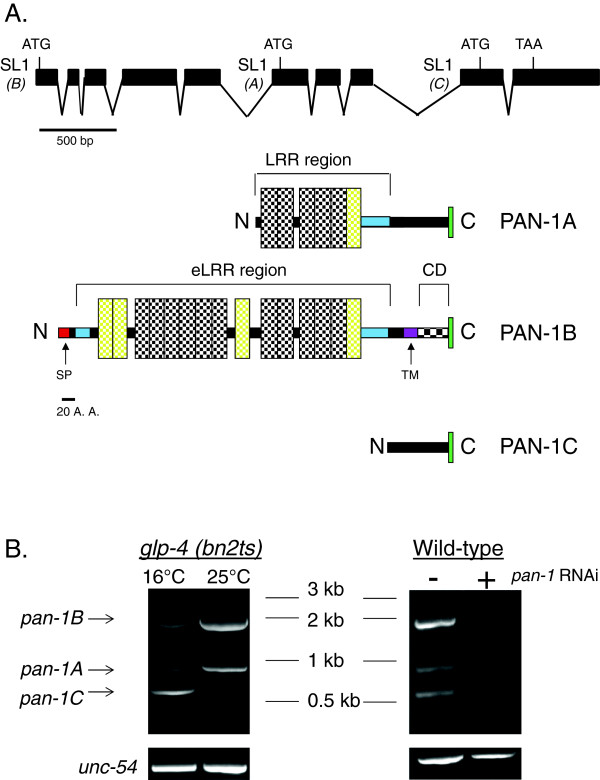
***pan-1 *gene structure and mRNA isoform expression. (A)** mRNA splicing pattern of *pan-1* and predicted encoded proteins. RT-PCR data indicate three *SL1 trans*-splice sites that would generate three distinct mRNA isoforms: *pan-1A, pan-1B,* and *pan-1C*. The isoforms generated by the *SL1 trans*-splice sites are indicated in parentheses. Possible translational start codons for the different isoforms are also indicated. The encoded PAN-1B protein isoform has 11 LRRs (vertical stippled boxes) as identified by the SMART program. An additional 4 LRRs are predicted by LRRscan, as described in Dolan et al. 2007 (yellow boxes). The N and C terminal LRR capping domains (blue colored regions) were identified using the consensus sequence found in Dolan et al., 2007. The PAN-1 N-terminal capping domain (LRR-NT) has the amino sequence CIDIEKGFKEEFNAHKQP VCICADNGIFSTVKGFTIEC. The C-terminal PAN-1 capping domain (LRR-CT1) has the amino acid sequence PWVCVCNDPKEWLPRWLEASEEADVAEGALGCLAIPNC. The signal peptide (‘SP’) is indicated by the red colored region and the transmembrane domain (‘TM’) is indicated by the purple colored region. The cytoplasmic domain (‘CD’) and GFP fusion point (green bar) are also indicated. PAN-1A is not predicted to encode a transmembrane protein and contains fewer LRRs. **(B)** RT-PCR analysis of *pan-1* on total RNA isolated from *glp-1(bn2ts)* and also wild-type adults subjected to control or *pan-1* RNAi (1% agarose gel stained with ethidium bromide). *glp-1(bn2ts)* larvae raised to adulthood at 16°C form a germline while animals raised at 25°C lack germline tissue. *pan-1A* and *pan-1B* are amplified from cDNA generated from both populations while *pan-1C* is only robustly amplified from cDNA generated from animals raised at 16°C, indicating germline enrichment of *pan-1C* relative to the soma. All three isoforms are also amplified from cDNA generated from wild-type adult animals. *unc-54* is a somatic gene that is expressed in both 16°C and 25°C cDNA preparations.

Transcript evidence in wombase.org indicates that two distinct mRNA isoforms are transcribed from the *pan-1* gene. The two isoforms differ by the location of the *SL1 trans-*spiced leader sequence (Figure 1A). The larger isoform, *pan-1B,* encodes a predicted type I transmembrane protein containing 15 eLRRs [3]. This isoform is confirmed by analysis of existing expressed sequence tags and by a full-length cDNA sequenced for this study (see Methods). Existence of the shorter isoform, *pan-1A*, is based on RNA-seq data [25] that has been incorporated into the wormbase.org annotations. This alternate *SL1 trans*-spliced isoform lacks the first five exons of *pan-1B* and would encode a protein with 6 LRRs and lacking a signal sequence; thus PAN-1A is not predicted to be membrane localized (Figure 1A). To confirm the existence of the *pan-1A* isoform, we performed RT-PCR using primers corresponding to *SL1* and the *pan-1* 3’UTR on total RNA isolated from adult animals. PAN-1 is known to be a component of P-granules [18] but it has not been determined which PAN-1 isoform is found in the germline and associated with P-granules. However, PAN-1A would be the most likely candidate since it is predicted to be cytoplasmic. To determine if any of the mRNA isoforms are enriched in the germline relative to the soma, we performed the RT-PCR analysis on *glp-4(bn2ts)* mutants. *glp-4* is essential for germline proliferation and the *glp-4(bn2ts)* allele is temperature-sensitive. *glp-4(bn2ts)* animals raised at 16°C produce a germline and are fertile while animals raised at 25°C are sterile due to a block in germline proliferation [26]. We detected three PCR products that were confirmed to be amplified from *pan-1* cDNA by sequencing (Figure 1B). In addition to detecting *pan-1A* and *pan-1B*, the RT-PCR experiments also revealed another isoform which we designate as *pan-1C*. *pan-1C* is a small mRNA generated by SL1 *trans*-splicing to exon 9. It is predicted to encode a 92 amino acid presumably cytoplasmic protein lacking leucine-rich repeats (Figure 1A). The *pan-1C* isoform appears to be enriched in the germline relative to the soma as it was robustly amplified from cDNA generated from adult *glp-4(bn2ts)* worms raised at 16°C but not from adult worms raised at 25°C.

BLASTP analysis did not identify primary PAN-1B sequence homology to other eLRR proteins outside of *Caenorhabditis* (data not shown). However, previously published bioinformatic analysis of eLRR proteins clustered PAN-1B with the ‘LRR-Tollkin’ class of eLRRs, suggesting structural homology with eLRRs from *Drosophila* and humans [3]. The ‘LRR-Tollkin’ class includes TLRs and other eLRR proteins; however, PAN-1 does not possess the signature cytoplasmic toll/interleukin 1 receptor (TIR) domain found in other Toll-like eLRRs and it was not identified as being involved in innate immunity in an RNAi-based screen of *C. elegans* eLRR genes [27].

### Loss of *pan-1* function causes larval developmental arrest

**Figure 2:**
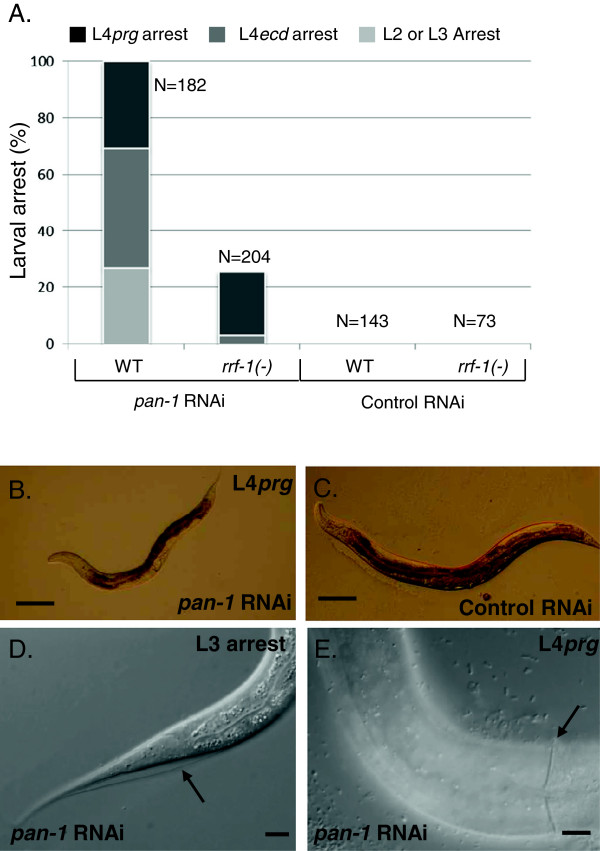
***pan-1 *is required for larval development. (A)** Graph showing the percentages of larval arrest phenotypes in *pan-1* and control RNAi experiments. L4*prg* and L4*ecd* larval arrest phenotypes are differentiated by a ‘clear’ appearance and un-molted pharyngeal cuticle in the L4*ecd* larvae. L4*prg* larvae appear healthy and are viable. **(B, C)** Representative micrographs of time-matched *pan-1* and control RNAi hermaphrodites (wild-type background). *pan-1(RNAi)* L4*prg* hermaphrodite is shown in **(B).** L4*prg* larvae are smaller than control animals **(C)** and sterile (no eggs generated). Scale bars = 100 μm **(D)** An arrested *pan-1(RNAi)* L3 larva encased in L2 cuticle (arrow). **(E)** An arrested L4*prg* hermaphrodite partially encased in the posterior segment of L3 cuticle (arrow). Posterior is to the right. Scale bars = 10 μm.

As observed in previous studies [18,19], we found that *pan-1* RNAi resulted in larval development defects associated with an inability to properly undergo ecdysis. Our experiments utilized the RNAi by feeding method [28,29] on L1 larvae hatched in the absence of food. In a wild-type background, *pan-1* RNAi caused larval arrest at different stages (Figure 2A-C). An incompletely penetrant L2 and L3 larval growth arrest was associated with an ecdysis defect where animals were completely encased in the previous stage cuticle, leading to death of the animal (Figure 2D). The remaining animals progressed to the L4 stage and were viable at the time of initial scoring (~54 hrs or later after RNAi feeding at 20°C) but failed to develop into reproductive adults. Most (58%) of the L4 arrested animals exhibited a ‘clear’ phenotype, or starved appearance, indicating feeding defects caused by improper molting of pharyngeal cuticle or complete encasement in previous stage cuticle (data not shown). The remaining (42%) of L4 arrested animals appeared healthy and active, although their movement was slightly uncoordinated and their size was smaller compared to control animals at the time of scoring (Figure 2B). Closer inspection of these animals revealed that they had partially shed cuticle that remained attached to the body (Figure 2E) which may account for their movement defect. However, these animals had molted their pharyngeal cuticle and were feeding. After an additional 20–24 hours of incubation, most of these healthier animals were still arrested in an L4-like state but remained viable and active. Thus, these animals represent a distinct L4 arrest phenotype marked by a failure to progress to the adult stage that is not solely due to an ecdysis defect. We refer to the arrested L4 animals with pharyngeal ecdysis defects as ‘L4*ecd*-arrested’ animals and the latter as ‘L4*prg*-arrested’ animals to indicate viable animals that fail to progress to the adult stage.

### *pan-1* function is necessary for development of the gonad and completion of vulval morphogenesis

**Figure 3:**
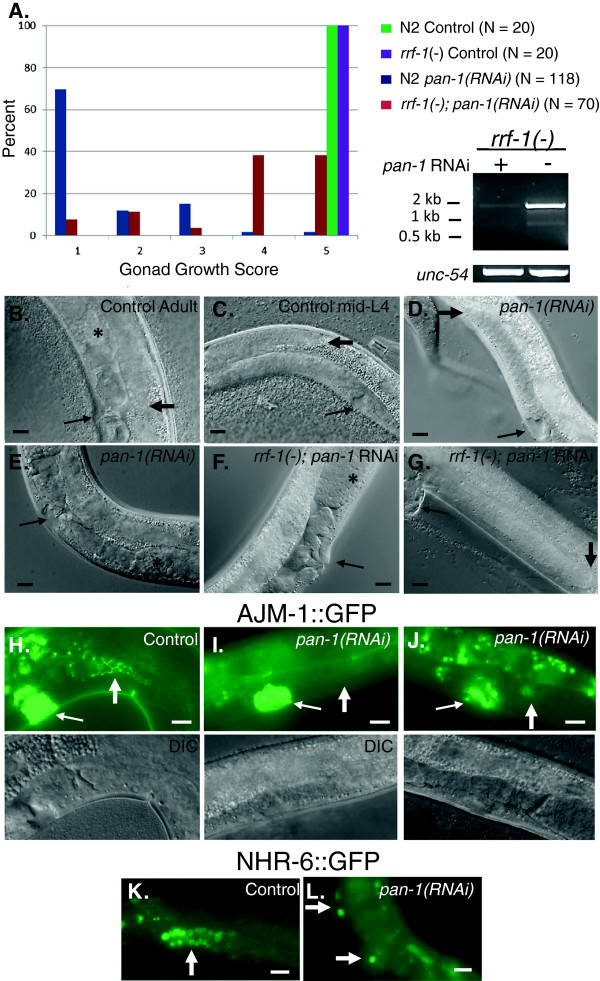
**Gonadal growth and vulva development phenotypes of *pan-1(RNAi) *hermaphrodites. (A)** Graph showing the gonadal growth scores of *pan-1* and control RNAi animals. The number of animals scored for each experiment is indicated. Also shown is RT-PCR analysis of *rrf-1(pk1417)* cultures (harvested at mid-L4 stage) subjected to control or *pan-1*RNAi. DIC micrographs of control RNAi **(B, C)** and *pan-1* RNAi **(D–G)** animals. In images **(B-G)**, the thin arrow indicates vulva, thick arrow indicates distal tip of gonad, and asterisk developing gametes. **(B)** Control RNAi hermaphrodite at 54 hrs post-feeding. **(C)** Control RNAi mid-L4 hermaphrodite (38–40 hrs post-feeding). Note luminal stage of vulval development and degree of gonadal growth. **(D)***pan-1(RNAi)* hermaphrodite at 54 hrs post-feeding exhibiting gonadal growth score of 1 and arrested vulva development at the luminal stage. **(E)***pan-1(RNAi)* hermaphrodite at 54 hrs post-feeding with a gonadal growth score of 3 (distal end not shown) and producing gametes. Vulva remains at the luminal stage of development. **(F)***rrf-1(−); pan-1(RNAi)* adult hermaphrodite exhibiting a normal vulval morphology and a morphologically abnormal but differentiated somatic gonad. **(G)***rrf-1(−); pan-1(RNAi)* adult hermaphrodite exhibiting a protruding vulva, a gonadal growth score of 1 and absence of somatic gonad tissue. Both of the animals in **(F)** and **(G)** completed the L4 to adult molt and synthesized lateral alae. Epifluorescence **(H-J)** and corresponding DIC micrographs of AJM-1:GFP expression in control **(H)** and *pan-1* RNAi hermaphrodites **(I, J)**. The animal in **(I)** exhibits an absence of spermathecal tissue (thick arrow) while the animal in **(J)** exhibits an abnormally formed and small spermatheca. Epifluorescence micrographs **(K, L)** of nuclear localized NHR-6:GFP expression in control **(K)** and *pan-1* RNAi hermaphrodites **(L)**. Normal spermathecae in late larvae and adults have 24 cells (thick arrow). In the *pan-1(RNAi)* animal one gonad arm has two expressing nuclei while the other has a single expressing nucleus. Scale bars = 10 μm.

Microscopic analysis of L4*prg-*arrested *pan-1(RNAi)* animals revealed severe defects in gonadal development. Control RNAi hermaphrodite animals had reached reproductive maturity when observed 54 hrs after RNAi feeding at 20°C. In contrast, *pan-1(RNAi)* hermaphrodites exhibited stunted gonadal growth. We devised a scoring system to quantify the gonadal defects in *pan-1* and control RNAi animals (Figure 3A). The most severe and frequent defect observed (score = 1) was growth arrest of both gonad arms prior to the distal tip of the gonad reflexing to the dorsal side of the animal (Figure 3B-D). Less penetrant and less severe defects observed included animals with one non-reflexed gonad arm and one reflexed but growth arrested gonad arm (score = 2); both gonad arms reflexed but growth arrested (score = 3); and a small percentage of animals with one normal sized gonad arm and one growth arrested gonad arm (score = 4) or two normal sized gonads arms (score = 5). Animals that exhibited the earliest gonadal growth arrest did not exhibit any evidence of gametogenesis or somatic gonad differentiation. Animals with less severe gonadal phenotypes initiated gametogenesis, although oocytes were only produced in a few animals (Figure 3E). Animals that achieved greater gonad growth also displayed more evidence of somatic gonad differentiation. However, sterility was fully penetrant.

To assess somatic gonad specification and differentiation in *pan-1 (RNAi)* hermaphrodites, we analyzed the expression of several somatic gonad markers. They included *ajm-1::GFP*; *nhr-6::GFP*[30], which is expressed in the spermatheca and dorsal uterine lineage and is thus a specification marker for these tissues; *lim-7::GFP*[31], which is expressed in the gonadal sheath cells beginning in L4; and *lag-2::GFP*[32], which is expressed in the distal tip cells*.* Expression of *ajm-1::GFP* was dependent on the degree of gonad growth in L4 arrested animals. Animals with the most severe gonadal growth phenotype (score = 1) did not express *ajm-1::GFP* (Figure 3H, I) indicating absence of spermatheca differentiation (n = 42). Expression of AJM-1::GFP was observed in animals with reflexed gonad arms, indicating spermatheca formation. However, all spermathecae in these animals were abnormally formed (Figure 3J). Absence of AJM-1::GFP expression was also observed in *ajm-1::GFP; pan-1 (RNAi)* animals scored at 78 hrs post-feeding that exhibited the most severe gonadal growth phenotype (n = 23).

A similar profile was observed with *nhr-6::GFP* but with some notable differences. A higher percentage (32%; n = 31) of *pan-1(RNAi)* animals with the most severe gonadal growth defect expressed *nhr-6::GFP* but only a small number of expressing cells were observed in these animals (range of 2–9 expressing nuclei) (Figure 3K, L). Thus, specification of spermathecal precursor cells can occur in the most severely affected animals. Consistent with other observations, a greater degree of spermatheca development was observed in animals with higher gonadal growth scores (data not shown). These animals formed two spermatheca primordia with each having a decreased number of cells (avg. =11 *nhr-6* expressing nuclei, n = 18) compared to the 24 cells/spermatheca in control animals. Similar results were observed with *lim-7::GFP.* This marker is expressed beginning in early-mid L4 stage and only *pan-1(RNAi)* animals containing reflexed gonad arms expressed *lim-7::GFP* (data not shown)*.* Even in animals that exhibited the most severe gonad growth phenotype, *lag-2::GFP* expression was not affected, indicating that distal tip cell specification is normal in these animals (Additional file 3: Figure S2).

A striking phenotype observed in *pan-1(RNAi)* animals was the inability of the vulva to complete later stages of morphogenesis. At the L3/L4 molt in wild-type animals the 22 cells comprising the developing vulva invaginate and begin to form a series of stacked toroids (vulA, B, C, D, E, F) with a vulval lumen [33] (see Figure 3C). By late L4 vulva development, the cells forming the toroid will homotypically fuse, attachments to the seam and vulval muscles are made, and a cuticle is secreted into the central lumen. The anchor cell also invades the apex of the invaginated vulva and fuses with a uterine cell, the utse. At the L4/adult molt, the vulva begins to evert so that the vulva slit is positioned outside of the animal (see Figure 3A). In *pan-1 (RNAi)* hermaphrodites, vulva invagination is initiated and proceeds to the luminal stage of development. However, vulva development does not progress past the luminal stage (Figure 3D, E). This defect was fully penetrant in a wild-type background and observed in most strains subjected to *pan-1* RNAi (see below). In addition, the same vulva defect was observed regardless of the level of gonadal development*.* The vulva did not progress past the luminal stage in RNAi animals that initiated gametogenesis and displayed differentiated somatic gonad tissue, even in animals scored at 74–78 hrs after dsRNA treatment (Figure 3E).

To further characterize the gonadal and vulva arrest phenotypes, we performed a time course analysis of *pan-1(RNAi)* animals in parallel to control RNAi animals. *pan-1(RNAi)* animals developed similarly to control animals through the L2 stage and both sets of animals had similar sized gonads at early L3 (28–30 hrs of development; n = 10 animals scored each for control and *pan-1* RNAi at each time point). However, by end of the L3 stage, developmental differences were apparent. At 32–34 hrs, control animals were transitioning to the L4 stage and gonad arms were initiating a dorsal turn while growth of the gonad arms in the *pan-1(RNAi)* animals had ceased. Gonad arms in *pan-1(RNAi)* animals were growth arrested (score =1) in all subsequent observations. At 44 hrs, 50% of the control animals were in the L4/adult molt and the other 50% at late L4 stage. All *pan-1(RNAi)* animals at this time point exhibited a vulva at the luminal stage of development. In addition, we scored 10 animals at the 54 hr time point with the most severe gonad phenotype and then scored these same animals 24 hrs later. Nine animals displayed no change in gonad growth indicating that this was a terminal arrest phenotype. The remaining animal had one gonad arm that had reflexed but remained growth arrested. The vulva in these animals remained arrested at the luminal stage and there was no evidence that the animals had initiated an additional molt. Thus, in the most severely affected *pan-1(RNAi)* animals there is a terminal developmental arrest of the gonad during the L3 stage and a terminal arrest of vulval development at the luminal stage.

We next asked if the gonadal arrest in *pan-1(RNAi)* animals reflected a germline or somatic function for *pan-1* in gonad development. To address this, we performed the RNAi experiments in an *rrf-1(pk1417)* mutant background. *rrf-1* mutants are defective in RNAi for most somatic tissues (epidermis, somatic gonad, muscle, neurons), but not the germline [34,35]. *pan-1* mRNA abundance was dramatically reduced in the experiments, most likely from RNAi knockdown in the germline (Figure 3A). As expected, *pan-1* RNAi in *rrf-1* mutants displayed a substantial decrease in the percentage of animals with molting defects (Figure 2A). Adult *rrf-1(pk1417); pan-1(RNAi)* animals produced adult alae and formed everted vulvae with both normal and abnormal morphologies (Figure 3F, G). A more complex result was observed for the gonad. The majority of *rrf-1(pk1417); pan-1(RNAi)* animals exhibited higher developmental gonad scores (Figure 3A). Most *rrf-1(pk1417); pan-1(RNAi)* animals exhibited evidence of somatic gonad differentiation, although only 12% of these animals generated morphologically normal somatic gonad tissues. The remaining animals had varying degrees of somatic gonad abnormalities. A brood size analysis also revealed that most *pan-1(RNAi); rrf-1(−)* animals were fertile (86.7%, n = 15). For fertile animals, brood sizes ranged from 1–124 (avg. 30.3 ± 38.8). In the few *rrf-1(pk1417); pan-1(RNAi)* animals that exhibited a gonadal development score of 1, there was no evidence of somatic gonad development (Figure 3G). Since the RNAi process can occur in the germline of *rrf-1* mutants, it would be expected that the gonadal phenotypes in *rrf-1(pk1417); pan-1(RNAi)* animals would be similar in severity to the phenotypes observed in a wild-type background if the essential functions for *pan-1* in gonadal development were solely in the germline.

The presence of gonadal phenotypes in the *rrf-1* experiments, albeit at lower penetrance and severity, indicates the L4*prg* phenotypes are not secondary effects from the partial ecdysis defect in these animals as *rrf-1(−); pan-1(RNAi)* animals are able to molt to the adult stage and synthesize alae.

### *pan-1* promotes development of the late larval hypodermis

**Figure 4:**
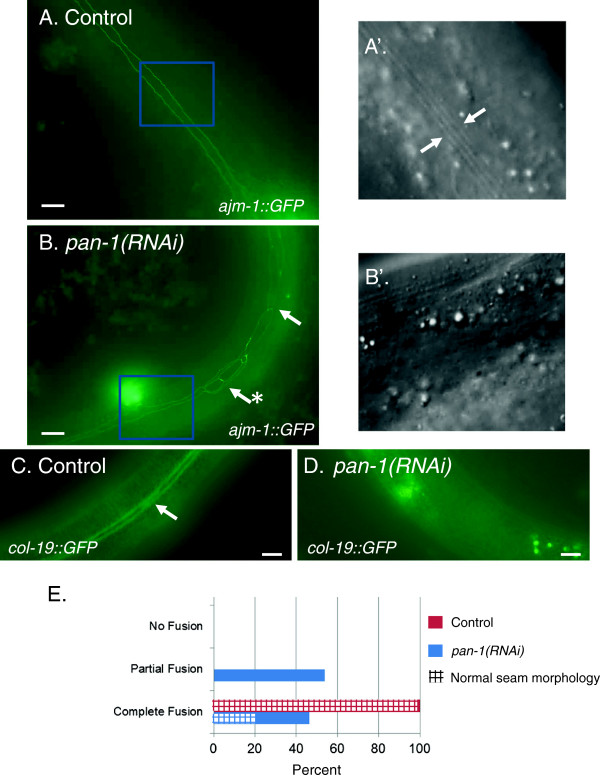
**Hypodermal defects of arrested *pan-1(RNAi) *animals.** Epifluorescence micrographs of *ajm-1::GFP***(A, B)** and *col-19::GFP***(C, D)** in control **(A, C)** and *pan-1* RNAi **(B, D)** animals. For **(A)** and **(B)**, the areas marked by the blue box are shown as enlarged DIC images **(A’ and B’)**. Animals subjected to *pan-1* RNAi do not synthesize alae (arrows in **A’**) in areas of normal seam fusion. The arrows in **(B)** represent areas of abnormal seam morphology including lack of fusion and abnormal morphology (arrow marked with an asterisk). **(C)** and **(D)***col-19::GFP* is not expressed in arrested *pan-1(RNAi)* animals. **(E)** Quantification of seam phenotypes in control (red bar) and *pan-1* (blue bar) RNAi animals. The stippling indicates the proportion of animals exhibiting completely normal seam morphology. N = 41 for *pan-1* RNAi; N = 20 for control RNAi. Scale bars = 10 μm.

It was also observed that L4*prg*-arrested animals failed to express markers associated with development of the hypodermis in the L4 stage. These animals did not have lateral alae, a cuticular structure synthesized at the L4 to adult molt (Figure 4A, B). This phenotype was fully penetrant. In addition, *pan-1(RNAi)* animals also did not express *col-19::GFP*. *col-19* encodes a cuticular collagen that is only expressed during the synthesis of adult cuticle (Figure 4C,D) [36,37].

The absence of alae synthesis and *col-19* expression is also observed in retarded heterochronic mutants [37,38]. At the L4 to adult transition, seam cells exit the cell cycle, fuse and synthesize alae. In retarded heterochronic mutants larval fates are reiterated, the seam cells fail to fuse at the L4 to adult transition and some retarded heterochronic mutants exhibit an increased number of seam cells due to reiteration early larval seam cell division patterns [39]. To determine if the seam cells of arrested *pan-1(RNAi)* animals exhibited features of a retarded heterochronic-type defect, we analyzed seam cell fusion using *ajm-1::GFP*. Seam fusions were observed in all arrested animals, although some animals had a mixture of partial and complete fusions (Figure 4E). Areas of the seam that fused failed to synthesize alae (Figure 4B). Abnormalities in the seam were also evident in most of the animals. Only 20.9% of animals exhibited complete seam cell fusions with an absence of any obvious abnormalities. Abnormalities included gaps and seam disorganization. With regard to seam cell number, we found that seam cell numbers were also not dramatically altered in *pan-1(RNAi)* animals (Additional file 4: Figure S3A). Normally, there are 16 seam cells at the L4 to adult molt. Using the *scm::GFP* seam cell marker [40], we found some deviation in seam cell number but the average remained close to 16. The fewest expressing cells observed was 11 and the most observed was 19. In animals with fewer cells, there appeared to be large gaps between SCM::GFP expressing nuclei (Additional file 4: Figure S3B, C). In many of these cases it appeared that a seam cell was present but not expressing SCM::GFP*.* Taking into account non-SCM::GFP expressing seam cells did not reveal any animals with dramatically altered seam cell number. Faint SCM::GFP expression was a common observation and worsened as the animals aged, preventing an accurate counting using SCM::GFP in older animals.

**Figure 5:**
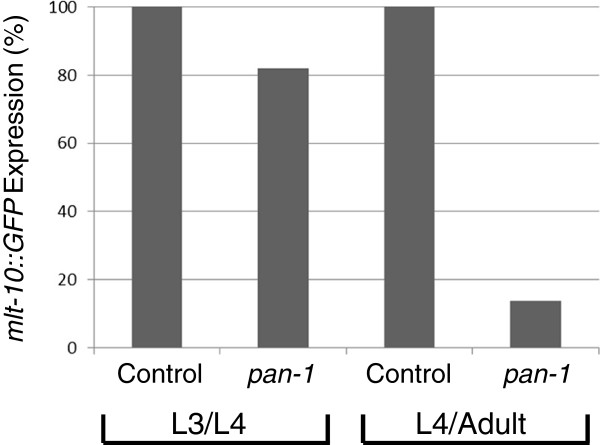
**Expression of *mlt-10::GFP*.** Percentage of animals expressing *mlt-10::GFP* at the L3/L4 transition (38-41hrs post-feeding) and L4/Adult transition (48–51 hrs post-feeding). N = 30 and 25 for control RNAi animals at L3/L4 and L4/Adult, respectively, and N = 33 and 36, respectively, for *pan-1* RNAi animals.

These results suggest that *pan-1* is not required for the temporal identity of the epidermis. The absence of alae and *col-19* expression, along with presence of partial seam cell fusion and malformed seam morphology, is more consistent with a failure of *pan-1* loss of function animals to properly progress through the L4 larval stage. To determine if *pan-1(RNAi)* animals initiate the L4 to adult molting process, we examined expression of *mlt-10::GFP* in *pan-1(RNAi)* animals. *mlt-10* encodes a proline-rich repeat protein that is proposed to function in the dynamic activities of cuticular proteins during cuticle synthesis and shedding [41]. *mlt-10* expression is initiated during latter part of the intermolt when genes with functions in cuticle synthesis and ecdysis are expressed. We found that *mlt-10::GFP* was expressed robustly in *pan-1(RNAi)* animals at the same time as control animals at the L3 molt (Figure 5; some *pan-1(RNAi)* animals at this time point failed to express *mlt-10::GFP* but these animals had not progressed through the L3 stage. Their numbers are included here as they were observed as part of a cohort that was time-matched to controls). As control animals were entering the L4 molt at 48 hrs post-feeding, the majority of time-matched *pan-1*(RNAi) animals were not expressing *mlt-10::GFP* (Figure 5). Expression was also not observed in the majority of animals with periodic observations (every hour) up to 60 hrs post-feeding. Non-expressing *pan-1(RNAi)* animals had the typical L4*prg*-arrest phenotype. A few animals did express *mlt-10::GFP* but these animals appeared to be progressing through the L4 stage. We noticed that *pan-1* RNAi was slightly less efficacious in the *mlt-10::GFP* strain as a small number adult escapers were consistently observed on RNAi plates. Thus, at the L4 stage, *pan-1* is necessary for *mlt-10* expression and normal progression of the molting process.

### *pan-1* expression pattern

To determine the expression pattern of *pan-1* we generated two different GFP reporter constructs. One construct is a transcriptional fusion containing 3 kb of sequence upstream of the predicted translation start codon (*pan-1prom::GFP*). This includes all of the 5’ sequence to the end of the nearest upstream gene. The other construct is a C-terminal fusion of GFP to the end of PAN-1 *(pan-1FL::GFP)*. This construct contained the entire *pan-1* genomic coding region and the 3 kb of upstream sequence. Three independent transgenic lines for each construct were examined and found to have identical expression patterns.

**Figure 6:**
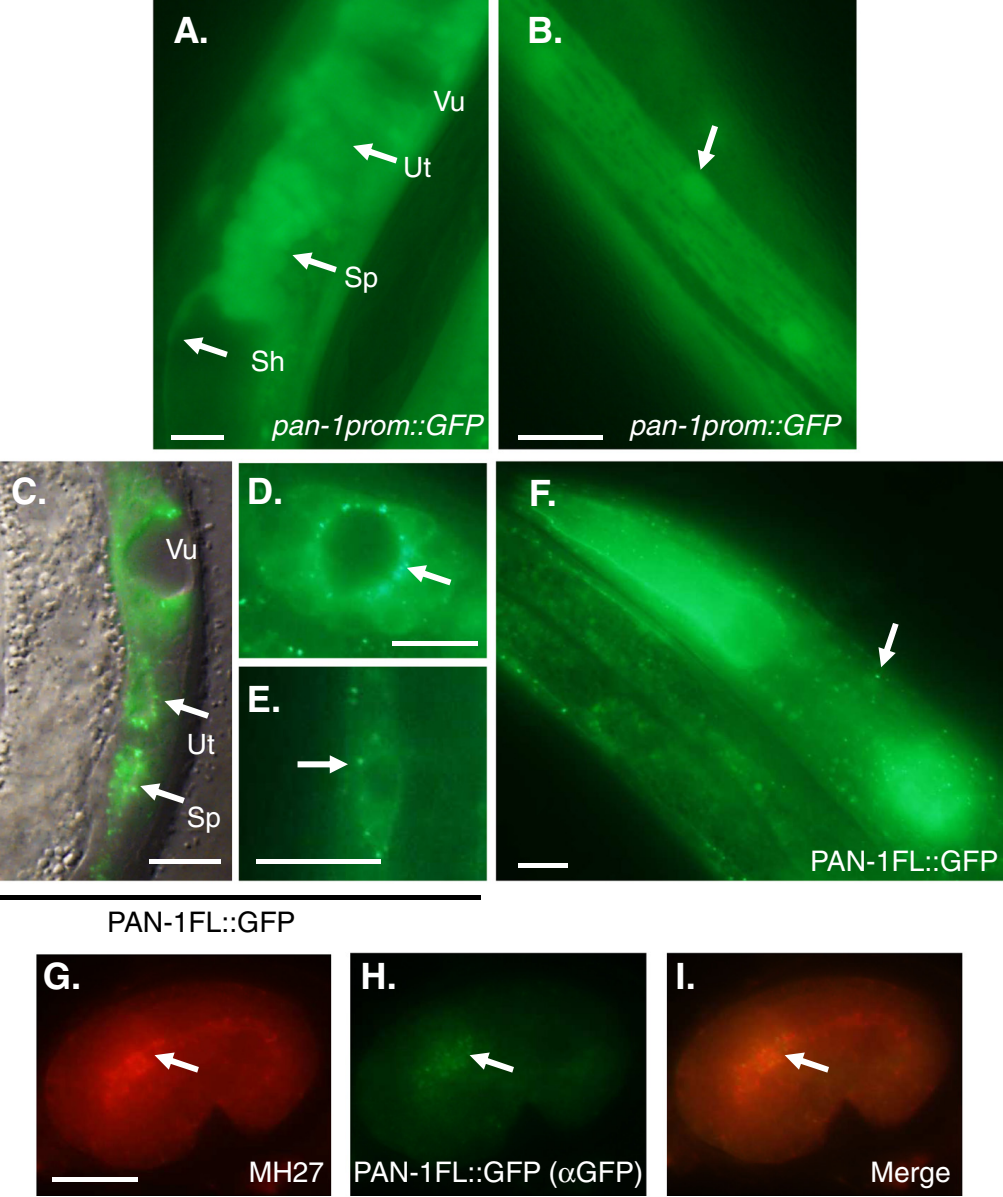
***pan-1::GFP *expression pattern. (A)** Expression of *pan-1prom::GFP* in the somatic gonad and vulva. The proximal sheath (Sh), spermatheca (Sp), and uterus (Ut) and vulva are indicated. **(B)***pan-1prom::GFP* expression in body wall muscle. Arrow indicates muscle cell nucleus. **(C)** A merged epiflourescence and DIC micrograph showing expression of PAN-1FL::GFP in the spermatheca, uterus, and vulva. The arrows indicate PAN-1FL::GFP punctae in the apical (lumenal surface) regions of spermatheca and uterine epithelia. **(D)** A ventral view of a developing L4 stage vulva showing PAN-1FL::GFP punctae clustered on the apical ends of vulva epithelial cells. Cytoplasmic fluorescence is also observed in these cells **(E)** PAN-1FL::GFP in a seam cell. Arrow indicates a punctate region of localization and cytoplasmic fluorescence is also observed. **(F)** Expression of PAN-1FL::GFP in the pharynx and anterior hypodermis. Cytoplasmic fluorescence is also observed in the pharyngeal tissue. Arrow indicates punctate localization in the hypodermis. Immunolocalization of MH27 **(G)** and PAN-1FL::GFP **(H)** in the developing pharyngeal epithelium. PAN-1FL::GFP is found in the developing pharyngeal epithelium (arrow) of a bean stage embryo. **(I)** Merged image of **(G)** and **(H)**. Scale bars = 10 μm.

Examination of transgenic animals bearing the transcriptional fusion revealed that *pan-1prom::GFP* is expressed in most somatic tissues. Expression was observed in all developmental stages from embryos undergoing morphogenesis to gravid adults. *pan-1prom::GFP* exhibited strong expression in body wall muscle, vulva, somatic gonad and pharynx (Figure 6). Expression was also observed in the nerve ring, hypodermis, and rectal epithelia. Expression in the intestine was typically not observed or very weak.

The *pan-1FL::GFP* transgene was able to rescue a *pan-1* deletion mutant. The *pan-1(gk142)* mutation is a 506 bp deletion that removes ~300 bp of sequence including the predicted translation start, the first exon and intron and some of the second exon. *pan-1(gk142)* homozygotes arrest as early larvae [18]. *pan-1(gk142)* homozygotes bearing the *pan-1FL::GFP* transgene develop normally to the adult stage and are fertile with wild-type level brood sizes (Avg. = 264 progeny; N = 4). With respect to spatial expression, the pattern of *pan-1FL::GFP* was the same as *pan-1prom::GFP* with some notable exceptions. First, PAN-1FL::GFP exhibited differences in expression intensity depending on the developmental point within the intermolt period. PAN-1FL::GFP was substantially qualitatively brighter at the mid to late intervals of the intermolt. Early in the intermolt bright expression of PAN-1FL::GFP was typically only observed in the pharynx. Expression was also weak in the adult with the pharynx being the predominant expressing tissue. This difference is likely due to perdurance of GFP in *pan-1prom::GFP* transgenics. In contrast, turnover of PAN-1FL::GFP would likely be similar to native PAN-1 protein. The other difference with respect to expression is that PAN-1FL::GFP was observed in the intestine, especially when expression was bright towards the end of the molt. This may indicate the presence of intestine enhancers within intron sequences of *pan-1.* PAN-1FL::GFP exhibited a punctate localization in expressing cells (Figure 6B, C, D E). In the vulva and somatic gonad epithelia (spermatheca and uterus), these punctae were concentrated on the apical (luminal) surface of these cells (Figure 6B, C). During embryonic pharyngeal development, PAN-1FL::GFP was found in the apical pharyngeal epithelium, as indicated by co-staining with the monoclonal antibody MH27 which recognizes the adherens junctions of epithelial cells (Figure 6G-I). The localization in apical regions of epithelial cells is consistent with membrane association of the PAN-1B isoform. Additionally, immunolocalization data for PAN-1 indicate membrane association in intestinal cells [18]. Cytoplasmic expression of GFP is also observed (likely corresponding to PAN-1A or PAN-1C); especially in the pharynx (Figure 6F) but also in other cell types (examples are shown in Figure 6D, E). However, apparent cytoplasmic expression was not consistently observed indicating that some cell types may only express cytoplasmic isoforms of PAN-1 at certain developmental times.

### Interactions with heterochronic genes

While the phenotypes described here would not classify *pan-1* as a heterochronic gene, *pan-1(RNAi)* animals do share phenotypic characteristics with retarded heterochronic mutants (absence of alae and partial seam fusion). Mutations in several heterochronic genes lead to precocious phenotypes where adult characteristics (such as alae) are expressed in larval stages. We therefore asked if precocious execution of adult fates in the heterochronic mutants would suppress the *pan-1* L4*prg*-arrest phenotype. *lin-28* and *hbl-1* are required for the execution of L2 fates and the deletion of L2 fates in *lin-28* and *hbl-1* mutants causes precocious alae formation at the L3/L4 molt [40,42,43]. As in wild-type animals *pan-1* RNAi blocked alae synthesis in *lin-28* and *hbl-1* mutants (Table 1). In addition to alae synthesis, other aspects of the *pan-1* L4*prg-*arrest RNAi phenotype were present in *lin-28* (−) and *hbl-1*(−) animals. *lin-28* and *hbl-1* mutants initiate vulval development precociously causing a protruding vulva (Pvl) phenotype at the L3/L4 molt. Most *hbl-1* and *lin-28* mutants subjected to *pan-1* RNAi were not Pvl and exhibited the arrested vulva development phenotype observed in *pan-1* RNAi experiments in a wild-type background. Thus, *pan-1* is necessary for the adult transition even when aspects of that process occur precociously.

**Table 1:** ***pan-1(RNAi) *interactions with heterochronic genes**

**Genotype**	**RNAi (N)**	**% With alae**	**% Arrested gonadal growth**^**1**^	**% Arrested vulva development**^**2**^
WT	*GFP* (10)	100	0	0
	*pan-1* (31)	0	88.7	100
*lin-28 (n719)*	*GFP* (15)	100	0	0
	*pan-1* (40)	5.0	100	100
*hbl-1(ve18)*	*GFP* (15)	100	0	0
	*pan-1* (38)	7.9	100	89.5^3^
*lin-29 (n333)*	*GFP* (15)	0	0	0
	*pan-1* (41)	0	85.4	14.6^3^
*let-7(n2853)*	*GFP*^4^	NA	NA	NA
	*pan-1* (30)	0	96.7	93.3

At least two independent RNAi experiments were scored and numbers combined. Experiments were performed at 20°C and phenotypes were scored 54 + hrs post-feeding, except for *let-7(n2853)* which was performed at 25°C and scored at 72 hrs post-feeding.

^1^Defined as gonad arms that failed to reflex. The gonad arms of hermaphrodites were scored independently (i.e. two gonad data points/animal).

^2^Defined as a vulva that invaginated but did not evert.

**Figure 7:**
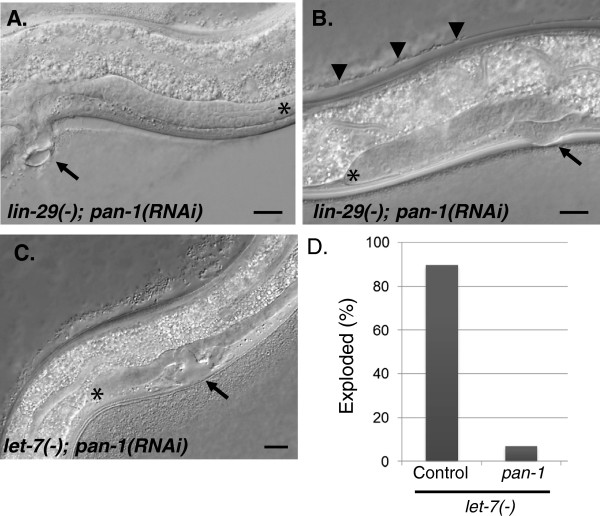
***pan-1 *RNAi interactions with *lin-29 *and *let-7*. (A)***lin-29 (−); pan-1(RNAi)* animal with a pronounced Pvl phenotype (arrow). **(B)***lin-29 (−); pan-1(RNAi)* animal with a smaller protruding vulva. Both animals in (**A**) and (**B**) exhibit an L3 gonadal arrest (*) and the animal in (**B**) is encased in unshed cuticle (arrowheads). **(C)***let-7(−); pan-1(RNAi)* animals with an L4 arrest phenotype marked by the developmentally arrested vulva (arrow) and stunted gonad (*). **(D)** The percentage of animals exhibiting the “exploded out of the vulva” phenotype. The *let-7* experiments were performed at 25°C as the *let-7(n2853)* allele is temperature-sensitive. The L4 arrest and exploded phenotype were scored at 74 hrs post-feeding. N = 126 for *pan-1* RNAi and N = 98 for control RNAi. Scale bars = 10 μm.

^3^Animals that had pronounced Pvls were not examined (see text). The animals that did not have arrested vulva development had small protruding vulvas (Figure 7B).

^4^These animals had “exploded out of the vulva” at the scoring time point and were not observed at high magnification (see Figure 7).

NA – not applicable.

The result was more complex with the retarded heterochronic gene *lin-29*. *lin-29* encodes a C_2_H_2_ zinc finger protein that functions at the end of the heterochronic pathway and is required for the execution of adult epidermal fates and cessation of molting [37]. *lin-29* mutants fail to cease the molting cycle at the fourth molt and do not synthesize alae. They also exhibit a Pvl phenotype that is partly due to a failure of the vulva to attach to the seam [44]. *pan-1* RNAi in a *lin-29 (−)* background generated an array of phenotypes distinct from *pan-1* RNAi in a wild-type background. Early larval and L4*ecd* arrest similar to *pan-1* RNAi in a wild-type background was observed in 33.8% of animals (n = 234). A pronounced Pvl phenotype was observed in 49.1% of animals (Figure 7A). Some of the Pvl animals had ecdysis defects and were completely encased in cuticle. Most of the animals that were not Pvl exhibited a smaller protruding vulva when examined at higher magnification (Table 1; Figure 7B). Therefore, unlike the observations with the precocious heterochronic mutants, *pan-1* RNAi did not completely prevent the formation of a protruding vulva in a majority of *lin-29* mutants. Since vulval eversion occurs at the fourth molt, the results are consistent with some *lin-29(−); pan-1(RNAi)* animals progressing through the L4 stage and executing a fourth molt with defective ecdysis occurring in some instances. An examination of late L4 *lin-29(−); pan-1(RNAi)* animals revealed that some had a vulva that developed past the luminal stage (11/20) while the remaining had vulvae that were still in the luminal stage of development (data not shown). It is possible that the smaller protruding vulvae arise when *lin-29(−); pan-1(RNAi)* animals molt without completing vulva development. The gonad phenotypes associated with *pan-1*loss of function were not dramatically altered, indicating that *pan-1* does not interact with *lin-29* in regulating gonadal growth (Table 1 and Figure 7A, B).Thus, *lin-29* loss of function appears to partially suppress the molting and vulval components of the L4*prg* phenotype of *pan-1(RNAi)* animals.

Given this interaction, we tested another retarded heterochronic gene, *let-7*, that functions upstream of *lin-29* in late larvae to regulate the adult transition. *let-7* encodes a conserved microRNA that represses heterochronic genes that promote early larval fates [45-47]. Like *lin-29, let-7* mutants do not synthesize alae and fail to cease the molting cycle at the fourth molt. *let-7* mutants have a distinct phenotype where they explode out of the vulva at the fourth molt [46]. In contrast to *lin-29, let-7 (−); pan-1(RNAi)* animals were indistinguishable from *pan-1* RNAi L4*prg*-arrested animals in a wild-type background and exhibited arrested vulval and gonadal development phenotypes (Table 1 and Figure 7C). In particular, the gonadal growth phenotype was often more severe in *let-7* animals (Figure 7C). *pan-1* also strongly suppressed the “exploded” phenotype of *let-7* mutants (Figure 7D), as would be expected with a failure to progress through the L4 larval stage. The specific molting interaction with *lin-29* suggests that these two genes function together to promote progression through the L4 to adult molting cycle.

## Discussion

In this study, we describe a functional characterization of *pan-1*, a gene identified in an RNAi screen for genes required for development of the spermatheca, a somatic gonad organ. We show that *pan-1* has essential roles in *C. elegans* development including molting and developmental progression of the vulva and gonad.

### Regulation of the molting process by *pan-1*

Our data confirm a role for *pan-1* in regulating the larval molting process. This observation has also been made in previously published studies [18,19]. However, we provide a more detailed characterization of its molting functions. In our RNAi studies, some of the molting phenotypes involve an inability to properly undergo ecdysis as *pan-1(RNAi)* animals fail to fully shed previous stage cuticle. A role for *pan-1* in ecdysis is also indicated by the requirement of *pan-1* for *mlt-10* expression during the L4 stage. In the post-embryonic *pan-1* RNAi studies described here, we fail to detect expression of a *mlt-10::GFP* reporter during the L4 stage. This absence of *mlt-10* expression correlates with the *pan-1* RNAi L4 arrest phenotype. This L4 arrest phenotype also suggests a role for *pan-1* in molting regulation that is broader than a specific role in ecdysis. We did not observe evidence of new ecdysis defects in subsequent observations of arrested animals and the arrested development of the vulva and gonad persisted in *pan-1(RNAi)* animals until animals began to die by a degradation process. These observations, taken together with the *mlt-10::gfp* expression data, suggest a general failure of *pan-1(RNAi)* animals to progress through the L4 stage and undergo the adult molt.

Interestingly, this L4 arrest phenotype is similar to the early larval arrest phenotype of *pan-1* mutants. While our study did not involve an analysis of *pan-1* mutant animals arrested at earlier stages, this molting phenotype has been characterized by others [18]. Animals homozygous for the *pan-1(gk142)* deletion arrest prior to the first larval molt. However, these animals remain alive for up to 8 days without further growth (a “forever young” Peter Pan phenotype [18]). Thus, the early larval arrest of *pan-1* mutants is not due to a failure in ecdysis (although the exact cause of death was not characterized in the referenced study). While we observed larval death associated with defects in L2/L3 and L3/L4 ecdysis in our post-embryonic RNAi experiments, these observations can be explained by *pan-1* having two molting functions: 1) progression through the larval stage and the initiation or execution of the molting process; and 2) a specific role in ecdysis. The failure to observe an earlier larval arrest similar to the L4 arrest in our experiments is likely due to post-embryonic RNAi failing to sufficiently reduce *pan-1* function in earlier larval stages. The data from both this study and previous *pan-1* studies suggest that *pan-1* functions throughout larval development, although we cannot exclude the possibility that *pan-1* may have functions in larval stage progression only during the L1 and L4 stages.

Other data support the hypothesis that *pan-1* promotes progression through the L4 stage. In particular, *pan-1(RNAi)* animals fail to synthesize adult alae and fail to express *col-19*, a gene encoding an adult-specific cuticle collagen. *col-19* expression and alae synthesis are also absent in retarded heterochronic mutants. However, the effect of *pan-1* loss of function is distinct from the heterochronic mutants. While the retarded heterochronic mutants fail to express adult-specific markers they still execute the molting process normally. In addition, other features of the larval to adult transition are affected in retarded heterochronic mutants, specifically the cell cycle exit and fusion of the seam cells. In contrast, *pan-1* loss of function animals have an average seam cell number similar to wild-type animals and seam cell fusion occurs to some degree in all *pan-1(RNAi)* animals. We therefore interpret the alae synthesis and *col-19* expression defects to be more consistent with a failure in L4 progression rather than a specific heterochronic-type defect in temporal specification, although it should be noted that some aspects of the retarded heterochronic phenotype are observed in *pan-1(RNAi)* animals, including modestly elevated seam cell number and partial failure of seam cell fusion in some animals. It is possible that *pan-1* activity intersects with both the molting and heterochronic pathways.

Interaction with the heterochronic pathway is further supported by *pan-1* RNAi experiments in a *lin-29* mutant background. *lin-29* loss of function appears to partially suppress the non-gonadal aspects of the L4*prg* phenotype of *pan-1(RNAi)* animals. The majority of *lin-29(−); pan-1(RNAi)* animals exhibit a protruding vulva, similar to *lin-29* single loss of function animals, indicating that *lin-29(−); pan-1(RNAi)* animals are to able execute the fourth molt. This apparent suppression of the *pan-1(RNAi)* L4*prg* phenotype could be due to an experimental anomaly or it represents a biologically relevant interaction with the *lin-29* gene. For the former, it is possible that *pan-1* RNAi is less efficacious in a *lin-29* mutant background. Arguing against this possibility is that the penetrance of the *pan-1* RNAi gonad phenotype is similar to *pan-1* RNAi in wild-type animals. One reason why the *pan-1* RNAi phenotype is not observed in *lin-29* mutants is that these animals are not undergoing a true larval to adult molt. Instead, *lin-29* mutants reiterate a larval-type molt at the fourth molt. This explanation would further support an important and distinct role for *pan-1* at the L4 to adult transition. However, the *pan-1* RNAi phenotype is not suppressed by mutations in *let-7,* which acts before *lin-29* in the heterochronic pathway. *let-7* mutants also reiterate a larval-type molt at the fourth molt. The *lin-29 (−)* heterochronic defect is not suppressed by *pan-1* loss of function (alae data, Table 1) and ecdysis defects are also observed in the double loss of function animals. Thus, it is possible that *pan-1* and *lin-29* have an interaction specific to the execution of fourth molting cycle but not in developmental timing or ecdysis. It is also interesting to note that computational analyses indicate the presence of an mRNA sequence within the *pan-1* 3’UTR that could bind *let-7* family member microRNAs [48,49]. However, the *pan-1* 3’UTR does not appear to have a critical function as the PAN-1FL::GFP fusion transgene that rescues *pan-1* mutants lacks the native *pan-1* 3’UTR.

### Role for *pan-1* in gonadal and vulval development

The proposed role for *pan-1* in larval stage progression is not limited to the hypodermis. Our experiments have a revealed a role for *pan-1* for both growth and migration of the gonad, the development of somatic gonad tissue that occurs in the L3 and L4 stages, and completion of vulval morphogenesis. Substantial growth of the gonad arms occurs in L3 and L4. The most severe phenotype was an arrest of gonadal growth in the L3 stage that persisted in *pan-1(RNAi)* arrested animals. This represented the most severe *pan-1(−)* gonadal growth phenotype with an absence of somatic gonad tissue and gametogenesis. In *C. elegans*, gonadal growth and migration is dependent on the function of the distal tip cell (DTC) [50]. We find that the gene *lag-2*, a marker for DTC fate and also a gene that functions in promoting germ cell proliferation, is expressed in *pan-1* RNAi animals, indicating formation and specification of the DTC. Up until the arrest of gonadal growth, we also do not see any overt effects on germ cell proliferation, suggesting that this particular DTC function may be normal. Experiments with cell markers for other somatic gonad tissues reveal a block in development prior to differentiation of these tissues in the most severely affected *pan-1(RNAi)* animals. Thus, *pan-1* is required for the developmental events that lead to the formation of a functional adult gonad. In this sense, the function of *pan-1* in the gonad is similar to its function in the hypodermis: promoting the progression of developmental events that lead to the differentiation of adult tissues. The somatic gonadal phenotypes of *pan-1(RNAi)* animals that escape L3 gonadal arrest and the phenotypes of animals from RNAi in the *rrf-1(−)* background suggest that *pan-1* functions through the entire duration of gonad development.

A major question is whether *pan-1* functions in the germline or soma in regulating gonadal growth. *pan-1* RNAi in *rrf-1(−)* mutants restored fertility and improved gonadal growth in a majority of animals. The *rrf-1(−)* RNAi results of our study produced a range of phenotypes similar to results described in the Gao et al. study [18]. Assuming that *pan-1* is strongly knocked down in the germline but not in the soma (as was determined in the Gao et al. study) it would suggest a role for *pan-1* in the soma in regulating gonadal growth and differentiation as it would be expected that the gonadal growth phenotype in the *rrf-1* experiments would be similar to RNAi experiments in the wild-type background if the critical *pan-1*function in this process were solely restricted to the germline. However, since compete loss of *pan-1* in the germline has not been verified in either study, it is difficult to assign tissue-specific roles to PAN-1 in regulating germline growth. PAN-1 does function in the germline based on its physical interaction with the germline RNA helicase, GLH-1 and its co-localization with P-granules in the germline and early embryo [18]. However, our results suggest that germline expressed *pan-1* may not be essential for fertility based on the *rrf-1* experiments and the observation that a transgene expressed PAN-1FL::GFP fusion fully rescues *pan-1* mutants. While we observe somatically expressed PAN-1FL::GFP we do not observe expression of PAN-1FL::GFP in the germline as would be expected due to the silencing of non-complex transgenes in the germline, although we cannot exclude the possibility that low levels of expression occur that escape our detection.

*pan-1* loss of function also causes a developmental arrest of the vulva. *pan-1 (RNAi)* animals initiate vulval development but fail to complete the morphogenetic process. This phenotype is also similar to the hypodermal and gonadal phenotypes in that it is another example of a failure to complete a L4 developmental process. Thus *pan-1* regulates the developmental progression of four distinct tissues: germline, somatic gonad, hypodermis, and vulva.

### Cellular role for *pan-1* in regulating epidermal and gonadal development

One surprising finding from this study is that the *pan-1* gene encodes both predicted transmembrane and cytoplasmic protein isoforms. We confirm that the *pan-1B* mRNA isoform is expressed and predicted to encode a type I transmembrane protein with the LRRs being positioned in the extracytoplasmic domain. Analysis of the PAN-1B protein with the TMHMM program (http://www.cbs.dtu.dk/services/TMHMM/) shows a high probability of a single transmembrane region at the C-terminal end of the protein. Our analysis of a full-length and functional C-terminal GFP-tagged PAN-1B fusion protein is also consistent with PAN-1B being membrane associated. This fusion protein is found in discrete punctae in all cell types where it is expressed. In addition, these punctae are concentrated in apical regions of epithelial tissues. Similar punctae have been observed for other fusions to transmembrane proteins, including eLRR proteins [16,17,41]. Therefore, PAN-1B is likely to function at the cell surface. PAN-1 is expressed in all of the tissues affected in our RNAi experiments, including the somatic gonad, hypodermis, and vulva. Our results also confirm the existence of the *pan-1A* mRNA isoform. The PAN-1A protein encoded by this mRNA would lack the signal sequence and would thus be cytoplasmic and not membrane associated. It would also have fewer LRR repeats thus potentially altering its protein-protein interaction partners. This protein isoform could be the isoform associated with P-granules in the germline. Interestingly, another predicted cytoplasmic PAN-1 protein isoform, PAN-1C, is encoded by the *pan-1* gene. We show evidence that the mRNA encoding this isoform is enriched in the germline relative to the soma making it another possible PAN-1 isoform associating with P-granules. It is possible that pan- 1C is only expressed in the adult germline as this isoform was not amplified in RT-PCR analyses performed on mid-L4 animals (see Figure 3A).

Further studies will be required to determine if the transmembrane, or cytoplasmic forms, of PAN-1 are essential for the somatic functions described here. However, the proposition that PAN-1B is a transmembrane protein with extracellular leucine-rich repeats leads to the intriguing hypothesis that PAN-1 may activate or modulate intracellular signaling cascades in response to interactions with extracellular signals. The eLRRs of PAN-1B could act as receptor for a specific signal, released in response to developmental or environmental cues that promote growth, or mediate interactions with the extracellular matrix or with other cells. Disruption of cell interactions with the extracellular matrix could dramatically affect tissue development and this could lead to the gonadal and vulva development phenotypes described here. Another possible role of eLRR proteins is in modulating other signaling receptors through sequestering or localizing specific signaling proteins. While the investigation of these possibilities will depend on future studies, PAN-1B represents an opportunity to define a potentially unique signaling system contributing to organismal development and evolution. While PAN-1B appears to be nematode-specific based on sequence, its important role in nematode development makes it an excellent model for identifying eLRR activities that may be broadly conserved in other organisms.

## Conclusions

We conclude from these studies that *pan-1* is required for molting, fertility and progression of the L4 to adult transition. We show that *pan-1* has essential functions in promoting gonadal growth and differentiation in the L3 and L4 stages. In addition, *pan-1* is required in the L4 stage for the completion of vulva development, the expression of adult hypodermal differentiation markers and execution of the L4 to adult molt. The molting and vulva development phenotypes are partially suppressed by a mutation in *lin-29* indicating that PAN-1 intersects with LIN-29 activity during these processes. *pan-1* exhibits complexity in expression as the gene encodes for both transmembrane and cytoplasmic isoforms. PAN-1 also exhibits a broad and dynamic expression pattern during larval development.

## Methods

### *C. elegans* strains

Strains were maintained and manipulated under standard conditions. All analyses were performed at 20°C, except where noted. The following strains were obtained from the *Caenorhabditis* Genetics Center for use in this study: N2 (Bristol); GR1373: *eri-1 (mg366)* IV; NL2099: *rrf-3 (pk1426)* II; SU93: *jcIs1 [ajm-1::GFP]* IV; VC235: *+/mT1* II; *pan-1(gk142)/mT1[dpy-10(e128)]* III; TP12: *kaIs12 [col-19::GFP]*; NL2098: *rrf-1 (pk1417)* I; RB798: *rrf-1 (ok589)* I; DG1576: *tnIs6 [lim-7::GFP]*; JK2049: *qIs19 [lag-2::GFP]* V; MT1524: *lin28 (n719)* I; RG559: *hbl-1 (ve18)* X; MT7626: *let-7 (n2853)* X; MT333: *lin-29 (n333)* II; SS104: *glp-4(bn2ts)* I. Other strains that were utilized, GR1395: *mgIs49 [mlt-10p::gfp-pest; ttx-3::gfp]* and JR672: *wIs54 [scm::GFP]*, were kindly provided by Alison Frand of the David Geffen School of Medicine at UCLA. Strain DN1 (*rrf-3(pk1426)* II; *jcIs1* IV) was constructed by crossing *rrf-3(pk1426)* II males to *vab-9 (e1744)* II; *jcIs1* IV hermaphrodites. F2 Rol non-Vab progeny were collected and animals that did not segregate *vab-9 (e1744)* II homozygotes in the F3 were used to establish lines that were tested for *rrf-3 (pk1426)* II homozygous phenotypes (Him, sterility at 25°C, and increased RNAi sensitivity). All experiments performed were approved by the University if Louisiana at Monroe Institutional Biosafety Commitee.

### RNAi screening

RNAi by feeding was performed essentially as described [51]. Bacterial clones were grown for 12-hours in LB broth containing 100 μg/ml ampicillin. The bacterial culture was added to NGM medium containing 100 μg/ml carbenicillin and 1mM IPTG and dsRNA expression was induced overnight at room temperature. To identify new spermatheca development genes, a two-stage screen was performed using a Chromosome III RNAi library (Source Bioscience). In the first stage of the screen, a synchronized population of RNAi-sensitized *eri-1(mg366)* L1 larvae were subjected to RNAi by feeding in 8-well plates and subsequently observed for reproductive defects. Phenotypes screened for included considerably reduced brood size or sterility and/or abnormal egg morphology. The latter phenotype can be indicative of abnormal ovulation that can occur due to defective somatic gonad development or function. This initial screen identified 190 genes (out of 2,402 genes in the Chromosome III library) that displayed reproductive phenotypes. The initial gene hits from the primary screen were repeated in triplicate along with a set of non-overlapping genes derived from wormbase.org that were annotated to have a sterile progeny (STP) phenotype. A secondary RNAi screen was then performed using *rrf-3 (pk1426* II*; jcIs1* IV strain (strain DN1) on 110 genes identified in the primary screen. DN1 is an RNAi-sensitized strain where AJM-1::GFP (encoded in the chromosomally integrated *jcIs1* transgene) is localized to the adherens junctions of spermathecal cells. This was utilized as a cellular marker to assess spermatheca morphology. A minimum of 10 nematodes were screened at the early young adult stage when the spermatheca in wild-type animals is elongated and not compressed by developing oocytes. 37/110 genes exhibited abnormal AJM-1::GFP expression in >70% of animals scored in an experiment. The 37 RNAi constructs were then sequenced to verify gene identity. Four genes (C23G10.8, *ttr-32*, Y47D3A.29, and H06I04.3) were represented by multiple RNAi constructs; therefore, 31 unique genes were identified by the screen.

### *pan-1* RT-PCR and sequence analysis

Synchronized populations of *glp-4(bn2ts)* were grown at 16°C and 25°C until the adult stage and harvested for RNA isolation. RT-PCR analysis on wild-type worms subjected to control or *pan-1* RNAi were grown at 20°C and harvested at the adult stage for RNA isolation. Worms were harvested at the mid-L4 stage for RT-PCR analysis of *rrf-1(pk1427)* cultures subjected to control or *pan-1* RNAi. Total RNA was isolated using the Trizol reagent (Life Technologies) according to manufacturer’s instructions. Reverse transcriptase reactions were performed on 300ng of total RNA using the Bio-Rad iScript Kit. PCRs were performed with 1 μl of cDNA at an annealing temperature of 55°C. *pan-1* was amplified for 35 cycles and *unc-54* for 28 cycles. The following primers were used for *pan-1* amplification: SL1*:* 5’-GGTTTAATTACCCAAGTTTGAG-3’; UTR: 5’-AGATCAGTGGTGGATACCTG-3’. The following primers were used for *unc-54* amplification: 5’-GGATTGGTCTGGACGATTTG-3’ and 5’-TACCTTGCCGGAGAAATCAC-3’. *pan-1* PCR products were gel purified and directly sequenced. We also obtained and sequenced a cDNA clone obtained from Open Biosystems and generated by the *C. elegans* ORFeome project [52]. Sequence analysis demonstrated that this cDNA is identical to GenBank accession NM_065524.3 (PAN-1B). eLRR sequences in PAN-1B were identified using the SMART program [53], in addition to those described in Dolan et al. 2007 [3].

### *pan-1* RNAi experiments

All *pan-1* RNAi by feeding experiments utilized the feeding clone obtained from Open Biosystems (RCE1182-9362659). Control RNAi experiments utilized a feeding clone containing *GFP* sequence (pPD128.110 (L4417, Addgene)). For GFP strains a feeding clone containing *her-1* (RCE1182-9360012, Open Biosystems) was utilized as a negative control. Bacteria were freshly streaked out onto ampicillin (100 μg/ml)/tetracycline (12.5 μg/ml) plates and colonies were cultured in 2 ml LB in a 15ml conical tube for 6–7 hrs at 37°C with shaking. Bacteria were spread onto NGM Lite plates containing 200 μg/ml ampicillin and 1mM IPTG and induced overnight at room temperature. Synchronized L1 larvae, produced by hatching eggs in the absence of food [54], were added to freshly induced bacterial lawns. All RNAi experiments were performed with a parallel negative control and *pan-1* RNAi in the wild-type N2 strain to ensure RNAi efficacy.

For the time course analysis, observations on 10 animals each for control and *pan-1*RNAi experiments were made every two hours beginning at 16 hrs post-feeding and ending at 44 hrs post-feeding when control animals were initiating the L4 to adult molt. Platings were staggered to eliminate the necessity of overnight observations.

### GFP reporter analysis and immunolocalization

The *pan-1prom::GFP* transcriptional fusion was generated by amplifying 3 kb of genomic sequence 5’ of the ATG start with hi-fidelity Phusion polymerase (New England Biolabs). The PCR product was cloned into *Sph*I and *Bam*HI sites of pPD95.67 (L2459, Addgene). For the full-length translational GFP fusion, the entire genomic coding sequence of *pan-1* consisting of 3kbp of 5’ sequence and the coding sequence to the stop codon was PCR amplified and cloned into the *Sph*I and *Age*I sites of pPD95.67 in frame with GFP coding sequences.

Transgenics were generated using microinjection of 10 μg/ml of reporter construct and 100 μg/ml of the *rol-6* transformation marker (plasmid pRF4). Three independent lines for both the transcriptional (*sgEx28-30*) and translational (*sgEx31-33*) fusions were generated. *sgEx31* was crossed into strain VC235 (*+/mT1* II; *pan-1(gk142)/mT1[dpy-10(e128)]* III). *pan-1(gk142)* homozygotes bearing the *sgEx31* transgene were recovered and the transgene was found to fully rescue *pan-1(gk142)* homozygotes. Verification of *pan-1(gk142)* homozygosity in this strain was confirmed by PCR amplification of the *gk142* deletion.

For immunolocalization of the PAN-1::GFP fusion protein and MH27 antigen in embryos, eggs were freeze-cracked in liquid nitrogen on poly-lysine slides and fixed in −20°C methanol for 20 minutes. Embryos were incubated with anti-GFP polyclonal antibodies (Life Technologies) and MH27 monoclonal antibody (Developmental Studies Hybridoma Bank) at 1:1000 and 1:500, respectively. Secondary reactions were performed with TRITC-labeled goat anti-mouse and FITC-labeled goat anti-rabbit (Life Technologies) at 1:1000 dilutions.

## Competing interests

The authors declare that they have no competing interests.

## Authors’ contributions

CRG designed the study, performed most of the experiments, and wrote the manuscript. TDK performed the RNAi screen and phenotypic characterizations of spermatheca development genes. Both authors read and approved the final manuscript.

## Supplementary Material

Additional file 1: Table S1 Spermatheca development genes identified in RNAi screen. Click here for file

Additional file 2: Figure S1 Representative spermatheca development phenotypes identified in the RNAi screen. Epifluorescence **(A, C, E)** and corresponding DIC micrographs **(B, D, F)** of control **(A, B),***C23G10.8***(C, D),** and *smc-4***(E, F)** RNAitreated DN1 young adult hermaphrodites. The arrow indicates spermatheca AJM-1:GFP localization and the arrowhead indicates AJM-1::GFP localization in the vulva. *C23G10.8* is an example of a spermatheca development gene with a defective morphogenesis phenotype while *smc-4* is an example of the “No AJM-1:GFP” expression phenotype. Click here for file

Additional file 3: Figure S2 Expression of *lag-2::GFP* in *pan-1(RNAi)* animals. Epifluorescence **(A)** and corresponding DIC **(B)** showing expression of *lag-2:GFP* in the distal tip cell (arrow) of a *pan-1(RNAi)* L4*prg* animal. Scale bar = 10 mm. Click here for file

Additional file 4: Figure S3 Seam cell phenotypes of *pan-1(RNAi)* animals. **(A)** Arrested *pan-1(RNAi)* animals do not exhibit a significant difference in seam cell numbers compared to control (p = .17; Student’s *t-*test). Error bars + S.D. **(B)** Seam cells expressing *scm::GFP* in control RNAi animals. Image shows a region of three seam cells on one lateral side of the animal. (**C)***scm:GFP* expression in arrested *pan-1(RNAi)* animals. Four seam cells (arrows) are captured in this image. One seam cell, indicated by the circle, does not express *scm::GFP*. The presence of the nonexpressing seam cell nucleus is shown in the corresponding DIC image **(C’)**. Scale bars = 10 mm. Click here for file
