# Supplementary material for: The C. elegans gene pan-1 encodes novel transmembrane and cytoplasmic leucine-rich repeat proteins and promotes molting and the larva to adult transition
Source: BMC Dev Biol. 2013 May 17;13:21. doi: 10.1186/1471-213X-13-21 (PMC3679943; doi:10.1186/1471-213X-13-21)
Supplement: Additional file 1: Table S1 — Spermatheca development genes identified in RNAi screen. [file 1471-213X-13-21-S1.docx]

Supplementary Table 1. Spermatheca development genes identified in RNAi screen

| Gene | Conservation^a^ | Description | Spermatheca Defect^b^ |
| --- | --- | --- | --- |
| *W06E11.1* | H, D, S | RNA polymerase III subunit | No AJM-1::GFP |
| *cct-5* | H, D, S | T-complex protein 1 subunit epsilon/Chaperonin | No AJM-1::GFP |
| *smc-3* | H, D, S | Cohesion subunit | No AJM-1::GFP |
| *smc-4* | H, D, S | Condensin subunit | No AJM-1::GFP |
| *pars-1* | H, D, S | Glutamyl-prolyl-tRNA synthetase | No AJM-1::GFP |
| *H06I04.3* | H, D, S | SAM-dependent rRNA methyltransferase | No AJM-1::GFP |
| *rps-12* | H, D, S | 40S ribosomal protein S12 | No AJM-1::GFP |
| *kle-2* | H, D | condensin-2 complex subunit | No AJM-1::GFP |
| *C23G10.8* | N | Unknown | Morphogenesis |
| *evl-14* | H, D, S | cohesion protein Pds5p | Morphogenesis |
| *dcn-1* | H, D, S | UBA-like ubiquitin ligase | Morphogenesis |
| *pan-1* | N | Extracellular leucine-rich repeat | Morphogenesis/ No AJM-1::GFP |
| *T04A8.6* | H, D, S | RNA-binding protein | Morphogenesis |
| *R02F2.7* | N | Unknown | Morphogenesis |
| *let-716* | H, D, S | rRNA processing protein RRP5 | Morphogenesis/ No AJM-1::GFP |
| *cyk-1* | H, D | Diaphanous homolog | Morphogenesis |
| *exos-9* | H, D, S | Exosome complex exonuclease RRP45 | Morphogenesis |
| *ttr-32* | N | Transthyretin-related | Morphogenesis |
| *tlk-1* | H, D, S | tousled-like serine/threonine protein kinase | Morphogenesis |
| *gop-2* | H, D, S | Conserved ATP binding protein | Morphogenesis/ No AJM-1::GFP |
| *him-10* | H, D, S | Kinetochore protein | Morphogenesis |
| *C16A3.4* | H, D, S | C2H2 zinc finger protein | Morphogenesis |
| *cgh-1* | H, D, S | DEAD-box RNA helicase | Morphogenesis |
| *plk-1* | H, D, S | Polo kinase | Morphogenesis/ No AJM-1::GFP |
| *unc-32* | H, D, S | vacuolar proton-translocating ATPase | Morphogenesis/No AJM-1::GFP |
| *rnr-1* | H, D, S | Ribonucleotide reductase | Morphogenesis/ No AJM-1::GFP |
| *Y47D3A.29* | H, D, S | DNA polymerase alpha catalytic subunit | Morphogenesis |
| *ZC395.4* | N | Unknown | Morphogenesis |
| *F09F7.3* | H, D, S | DNA-directed RNA polymerase III subunit | Morphogenesis |
| *tbb-1* | H, D, S | Beta-tubulin | Morphogenesis |
| *ani-1* | H, D | Annillin | Morphogenesis |

^a^H=*Homo sapiens;* D=*Drosophila melanogaster;* S=*Saccharomyces cerevisiae;* N=nematode-specific; BLASTP with E-value < -10 was used to identify protein conservation.

^b^Spermathecae scored as “No AJM-1::GFP” lacked detectable AJM-1::GFP expression indicating absence of spermathecae (AJM-1::GFP expression was observed in other tissues, see Fig.1). This defect is likely resulting from spermatheca cell specification, cell proliferation, or severe cell differentiation defects. Spermathecae scored as “Morphogenesis” exhibited AJM-1::GFP expression in the spermatheca but the organ did not form correctly. Both phenotypes were sometimes observed in the animals scored for a particular RNAi experiment.
