# Supplementary figures and images for: The C. elegans gene pan-1 encodes novel transmembrane and cytoplasmic leucine-rich repeat proteins and promotes molting and the larva to adult transition
Source: BMC Dev Biol. 2013 May 17;13:21. doi: 10.1186/1471-213X-13-21 (PMC3679943; doi:10.1186/1471-213X-13-21)

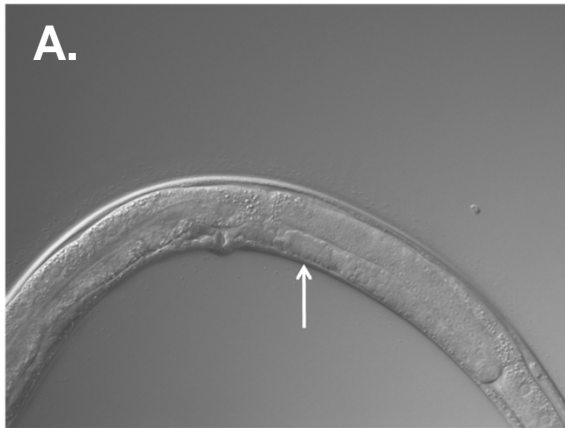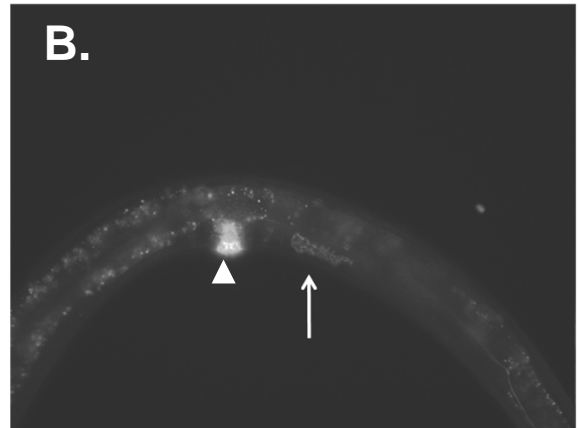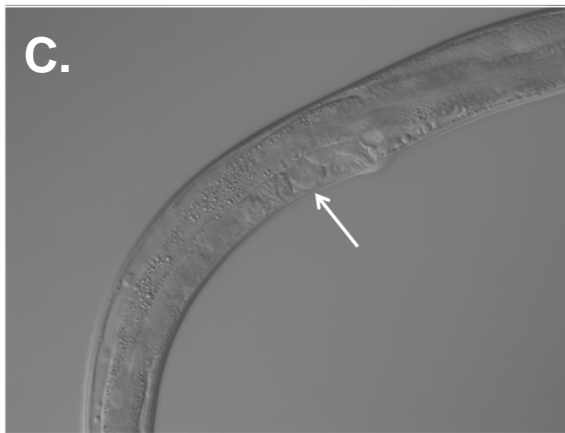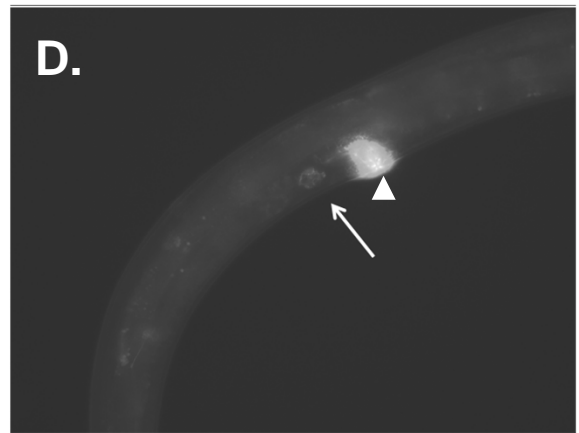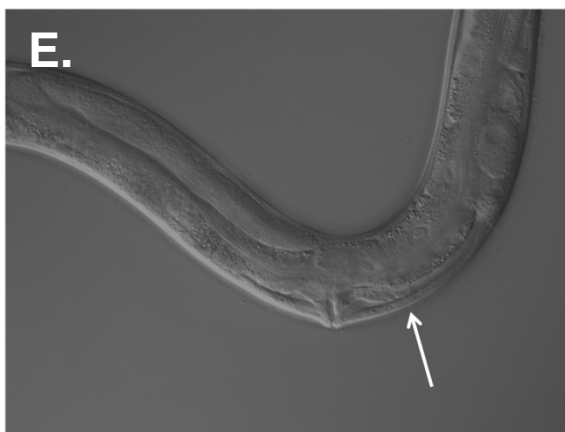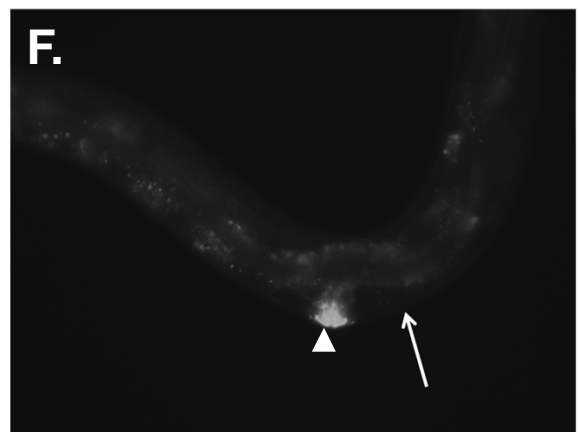

Supplement: Additional file 2: Figure S1 — Representative spermatheca development phenotypes identified in the RNAi screen. Epifluorescence (A, C, E) and corresponding DIC micrographs (B, D, F) of control (A, B),C23G10.8(C, D), and smc-4(E, F) RNAitreated DN1 young adult hermaphrodites. The arrow indicates spermatheca AJM-1:GFP localization and the arrowhead indicates AJM-1::GFP localization in the vulva. C23G10.8 is an example of a spermatheca development gene with a defective morphogenesis phenotype while smc-4 is an example of the “No AJM-1:GFP” expression phenotype. [file 1471-213X-13-21-S2.pdf]

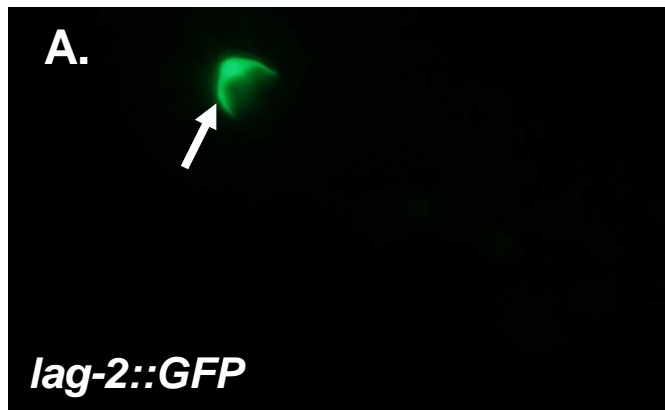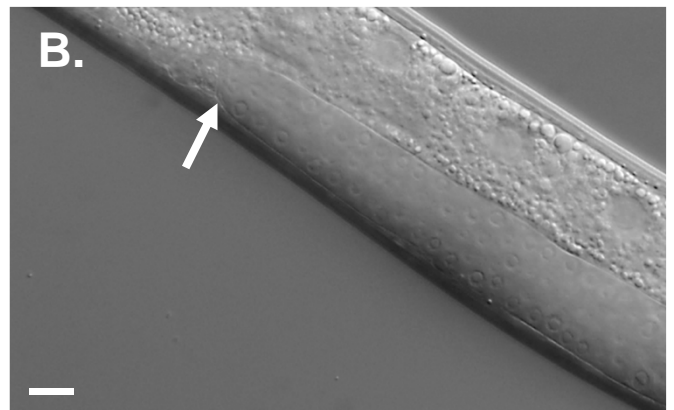

Supplement: Additional file 3: Figure S2 — Expression of lag-2::GFP in pan-1(RNAi) animals. Epifluorescence (A) and corresponding DIC (B) showing expression of lag-2:GFP in the distal tip cell (arrow) of a pan-1(RNAi) L4prg animal. Scale bar = 10 mm. [file 1471-213X-13-21-S3.pdf]

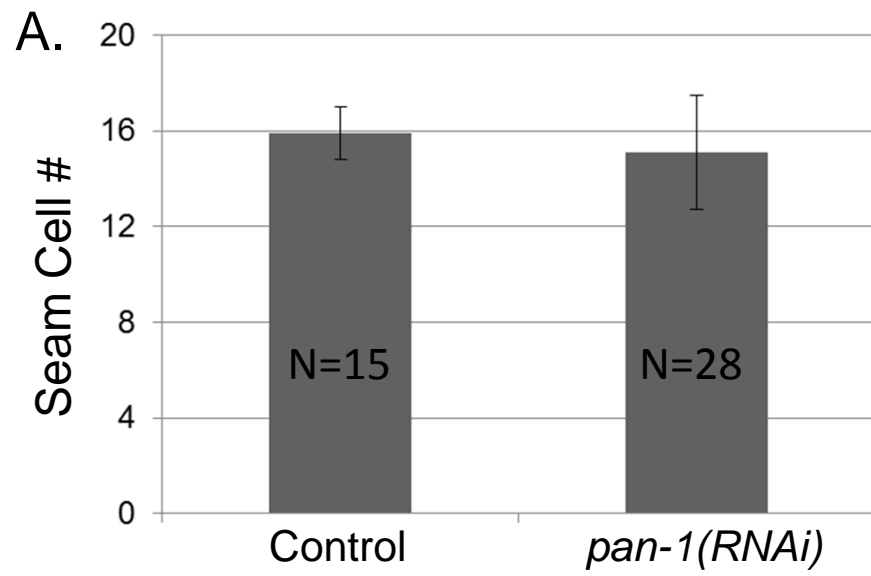

B. Control

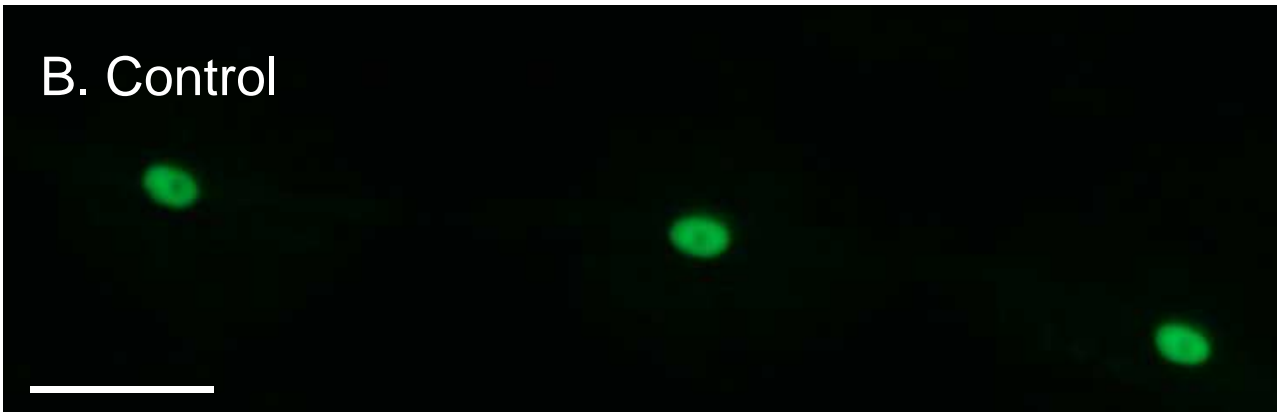

C. *pan-1(RNAi)*

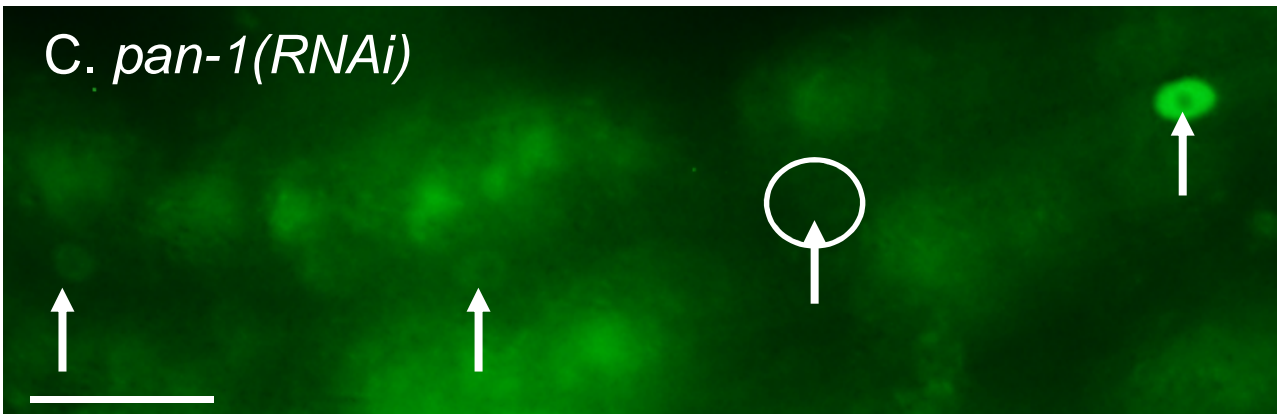

C'. *pan-1(RNAi)*

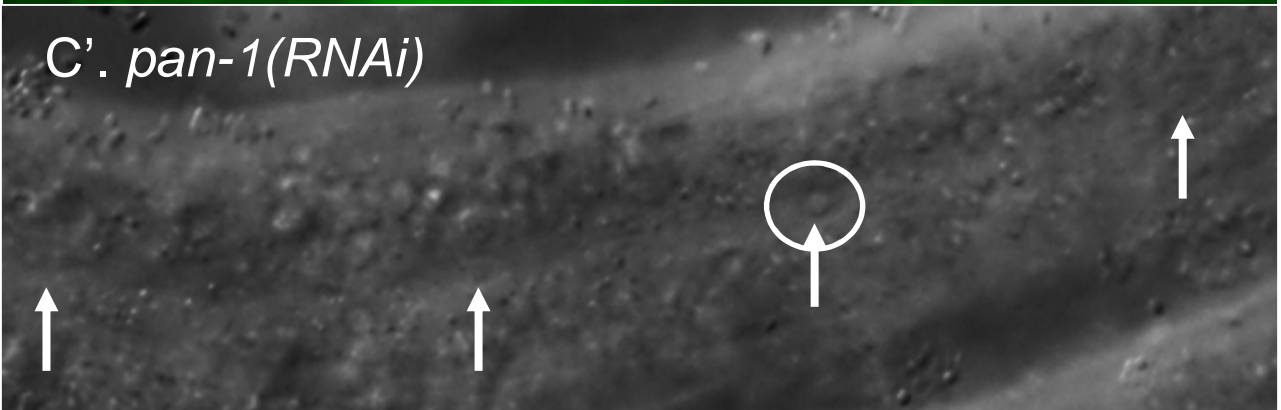

Supplement: Additional file 4: Figure S3 — Seam cell phenotypes of pan-1(RNAi) animals. (A) Arrested pan-1(RNAi) animals do not exhibit a significant difference in seam cell numbers compared to control (p = .17; Student’s t-test). Error bars + S.D. (B) Seam cells expressing scm::GFP in control RNAi animals. Image shows a region of three seam cells on one lateral side of the animal. (C)scm:GFP expression in arrested pan-1(RNAi) animals. Four seam cells (arrows) are captured in this image. One seam cell, indicated by the circle, does not express scm::GFP. The presence of the nonexpressing seam cell nucleus is shown in the corresponding DIC image (C’). Scale bars = 10 mm. [file 1471-213X-13-21-S4.pdf]
